# Integrated Approach to Eco-Friendly Thermoplastic Composites Based on Chemically Recycled PET Co-Polymers Reinforced with Treated Banana Fibres

**DOI:** 10.3390/polym14224791

**Published:** 2022-11-08

**Authors:** Martial Aime Kuete, Pascal Van Velthem, Wael Ballout, Bernard Nysten, Jacques Devaux, Maurice Kor Ndikontar, Thomas Pardoen, Christian Bailly

**Affiliations:** 1 Institute of Condensed Matter and Nanosciences—Bio &Soft Matter (IMCN/BSMA), UCLouvain, 1348 Louvain-la-Neuve, Belgium; 2 Macromolecular Chemistry Unit, Applied Chemistry Laboratory, Faculty of Science, University of Yaoundé I, Yaoundé P.O. Box 812, Cameroon; 3 Institute of Mechanics, Materials and Civil Engineering, UCLouvain, 2 Place Sainte Barbe, 1348 Louvain-la-Neuve, Belgium

**Keywords:** PET, recycling, glycolysis, solid-state polymerisation, composite, sustainability

## Abstract

A major societal issue of disposal and environmental pollution is raised by the enormous and fast-growing production of single-use polyethylene terephthalate (PET) bottles, especially in developing countries. To contribute to the problem solution, an original route to recycle PET in the form of value-added environmentally friendly thermoplastic composites with banana fibres (*Musa acuminata*) has been developed at the laboratory scale. Banana fibres are a so far undervalued by-product of banana crops with great potential as polymer reinforcement. The melt-processing constraints of commercial PET, including used bottles, being incompatible with the thermal stability limits use of natural fibres; PET has been modified with bio-sourced reactants to produce co-polymers with moderate processing temperatures below 200 °C. First, commercial PET were partially glycolyzed with 1.3-propanediol to produce co-oligomers of about 20 repeating units, which were next chain extended with succinic anhydride and post-treated in a very unusual “soft solid state” process at temperatures in the vicinity of the melting point to generate co-polymers with excellent ductility. The molar mass build-up reaction is dominated by esterification of the chain ends and benefits from the addition of succinic anhydride to rebalance the acid-to-hydroxyl end-group ratio. Infra-red spectroscopy and intrinsic viscosity were extensively used to quantify the concentration of chain ends and the average molar mass of the co-polymers at all stages of the process. The best co-polymers are crystallisable, though at slow kinetics, with a *T_g_* of 48 °C and a melting point strongly dependent upon thermal history. The composites show high stiffness (4.8 GPa at 20% fibres), consistent with the excellent dispersion of the fibres and a very high interfacial cohesion. The strong adhesion can be tentatively explained by covalent bonding involving unreacted succinic anhydride in excess during solid stating. A first approach to quantify the sustainable benefits of this PET recycling route, based on a rational eco-selection method, gives promising results since the composites come close to low-end wood materials in terms of the stiffness/embodied energy balance. Moreover, this approach can easily be extended to many other natural fibres. The present study is limited to a proof of concept at the laboratory scale but is encouraging enough to warrant a follow-up study toward scale-up and application development.

## 1. Introduction

Polyethylene terephthalate (PET) is a widely used thermoplastic polyester exhibiting attractive properties at reasonable cost, including low density, transparency, moderate chemical resistance, high tensile strength, melt processability and good gas barrier properties (e.g., against CO_2_) when biaxially oriented [1,2]. PET is used in many fields including construction, medicine, electronics, textile, automotive, aerospace and prominently in packaging [3,4]. Applications in packaging are dominated by single-use beverage containers, especially for mineral water and carbonated and energy drinks. Production has exploded during the last decades [4]. In 2021, the global consumption of PET bottles was estimated at about 19.8 Mt, with an average growth rate of 3.6% between 2012 and 2020 [5]. According to a recent forecast, 22.6 Mt of PET will be produced in 2025, good for a staggering 585 billion bottles [6,7]. The enormous and fast-growing production of single-use PET bottles obviously raises the major issue of disposal and environmental pollution, especially in developing countries where the waste recycling sector is underdeveloped or non-existent. The issue is compounded by the very high resistance of PET to environmental degradation [8]. PET accounts for a third of the plastic waste volume and ranks second for sea bed microplastic pollution [9]. These issues have recently attracted a lot of negative public attention and even outrage. On the other hand, PET is very recyclable and is already one of the most recycled thermoplastics [8]; however, the sheer size of the problem requires an even greater focus. It is essential to explore and develop new routes to valorise PET waste, which should be considered as an abundant and attractive material resource in its own right. As a matter of fact, this priority is already included in specific international legislation focusing on improved sustainability [7,10].

Various mechanical and chemical recycling routes have been developed and are continuously improved upon to valorise plastic waste, including PET. Mechanical recycling methods are currently the most used in industry and are based on melt reprocessing of the material after the life cycle of the original product. Unfortunately, the properties of such recycled materials are reduced by approximately 30% compared with those of virgin PET [11]. This “downcycling” characteristic of PET mechanical reconversion constrains the potential applications because the resulting cost–performance balance is inadequate. Di- and multifunctional chain extenders (e.g., epoxy-acrylate resins, di-isocyanates, etc., [12]) can be used during reactive extrusion of PET waste to compensate for molecular weight degradation due to reprocessing [13].

Chemical recycling is a “high end” alternative to mechanical recycling of PET as it delivers clean monomers or oligomers that can be incorporated in the synthesis of new value-added materials; possibly solving the cost–performance issue of mechanical recycling [7]. As an example, biodegradable aliphatic ester segments inserted between PET units can produce aromatic/aliphatic co-polyesters with good physical properties but improved biodegradability [14]. Indeed, the reduced crystallinity and faster degradation resulting from the presence of aliphatic co-monomers can improve degradation of (microplastic) waste by hydrolysis [9]. One important challenge of chemical recycling is to limit the environmental costs.

Beyond the straightforward recovery of the starting PET through a mechanical or chemical process, it is highly desirable to improve the property balance of the recycled materials, i.e., “upcycling” them by imparting additional features, in particular improved stiffness and strength at minimum environmental impact. Composites of a PET matrix with reinforcing fibres are well known. A wide range of fibres can be used and their detailed characteristics control the property balance of the end product, with glass and carbon as classical choices for improved stiffness and strength [15,16]. Natural fibres are an attractive choice for composites based on recycled polymers from the standpoint of sustainability. Among natural fibre candidates for incorporation into thermoplastic composites, certain types have received significant attention, in particular flax, jute and bamboo [17], whereas others are mostly neglected despite abundant and low-cost availability in extensive geographical areas. Banana fibres are a good example of the latter. They are extracted from the pseudo stems of the banana plant (*Musa acuminata*), which produces fruits only once and is treated as no-value waste in Equatorial and West African countries such as Cameroon and the Ivory Coast that have large banana plantations [18]. The wide availability, very low cost and attractive properties of the fibres after a simple treatment [19] make them great candidates for the development of environmentally friendly thermoplastic composites. The combination of banana fibres with recycled PET in the form of composites in banana-producing countries has the potential to contribute significantly to the mitigation of the plastic waste issue and the simultaneous valorisation of this so-far neglected crop waste.

However, the association of natural fibres, including banana fibres, and a PET matrix by melt processing at typical temperatures is very challenging due to the limited thermal stability of the fibres above 200 °C and the melt-processing temperatures in excess of 280 °C required by PET [20,21]. Consequently, working with banana fibres as reinforcement in PET composites requires the melt-processing temperature of the matrix to be reduced to the 200–220 °C range. In this work, we have overcome this incompatibility by chemically modifying the recycled PET to lower its melt-processing temperature by reducing its melting point, *T_m_*, and glass transition temperature, *T_g_*, within acceptable limits for potential applications.

**Figure 1:**
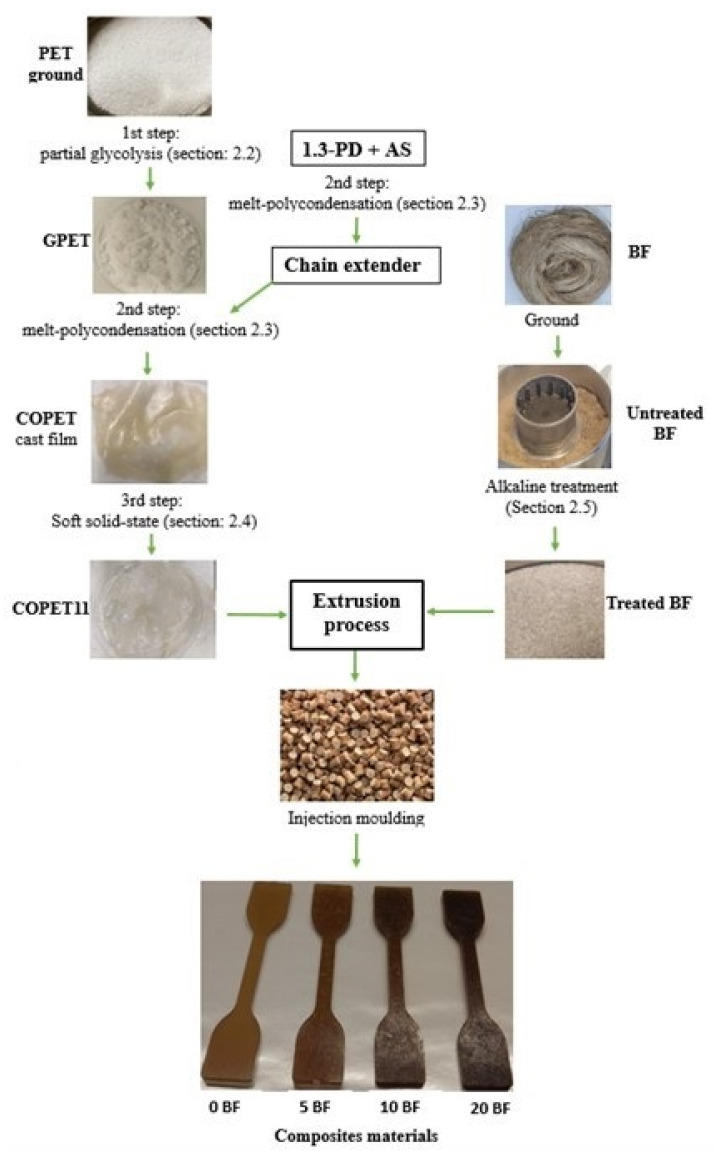
Schematic of PET recycling and composite processing developed in this work.

The original PET chemical recycling route is based on successive reaction steps in the melt or slurry and very unusually in the “soft” solid-state. It is carried out in simple reactors and ovens with no or little solvent added (easily recoverable). Only bio-sourced polymer co-units are used (1.3-propanediol and succinic anhydride). The first step is a partial glycolysis to yield PET oligomers of about 20 repeat units. The partial character of the glycolysis is a distinguishing feature of our process from the usual full glycolysis reported in the literature. It has the advantage of preserving as much as possible of the embodied energy of the starting polymer (i.e., the energy spent to produce it from crude oil) and thus reinforces the eco-friendliness. The second step is a condensation in the melt with the incorporation of bio-sourced co-units to reduce the melt-processing temperature of the resulting co-polymer. The third step is a molar mass build-up of the co-polymer to restore good mechanical properties by a very original “soft solid-state polycondensation” (S^3^P), which takes place in a quasi-molten state. In solid-state polymerisation (SSP), the diffusion of polymer segments and of end-groups is restricted to the sole amorphous phase [22] but the low crystallinity of the co-polymers minimises this restriction in our case. A schematic summarizing the steps involved in the polymer chemical recycling as well as the composite melt processing and testing is presented in Figure 1.

In addition, mechanically separated banana fibres are simply treated with sodium hydroxide in solution to improve their thermal stability and to optimise the cohesion with the matrix [23]. The co-polymer and treated fibres are finally melt-mixed in an extruder and shaped by injection moulding. Micrographs of fibre bundles (untreated and treated) as well as copolymer and composite test specimens are also shown in Figure 1.

In summary, we have contributed to solving the major environmental issue raised by PET single-use bottles by developing an original route to recycling PET. This is so far limited to a laboratory-scale proof of concept in the form of “upcycled” environmentally friendly thermoplastic composites with banana fibres; an undervalued by-product of banana crops with a great potential as polymer reinforcement. The approach is based on PET chemical modification with bio-sourced reactants in an original combination of steps (see Figure 1). It overcomes the incompatibility between the melt-processing constraints of commercial PET and the thermal stability limits of the banana (and all other) natural fibres.

The copolymers and the resulting composites were thoroughly characterized and tested. Moreover, an elementary approach to assess the sustainability of this PET recycling route, based on a rational eco-selection method, confirms the promise of our approach.

The potential impact of this work goes well beyond the PET–banana fibre composites studied since the PET modification developed here can be used to prepare composites with many different natural reinforcing fibres with comparable thermal stability and surface characteristics. Because this study focuses on a proof of concept, no attempt was made to further optimise the process kinetics and workup steps. This is left to a future study and is admittedly key to validate the economic feasibility of the concept and thus the application potential.

Although virgin PET as a starting material was used for convenience instead of polymer from recycled bottles, this does not reduce in any way the applicability of the concept to recycled PET. Several studies have indeed demonstrated that the environmental degradation of PET (e.g., used bottles exposed to the sun and rain even for a long period) by hydrolysis as well as UV degradation is limited to a thin layer at the exposed surfaces [24]. The high resistance of PET to environmental degradation is an advantage for recycling. As will become clear in the Section 3, the vital information needed for successful co-modified PET synthesis is the knowledge of the OH and COOH chain end concentrations after the glycolysis step.

## 2. Materials and Methods

### 2.1. Materials

The polyethylene terephthalate (PET) grade was Lighter C88 from Equipolymer (Amsterdam, The Netherlands), as used for plastic bottles (density 1.28 g/cm^3^ and *M_n_* = 28,500 g/mol). This highly crystalline material (obtained by solid-state polymerisation) was first extruded to obtain amorphous pellets (material from recycled bottles is also amorphous albeit highly oriented) and ground for subsequent depolymerisation. Propane-1,3-diol (C_3_H_8_O_2_), zinc acetate (Zn(CH_3_COO)_2_, antimony oxide (Sb_2_O_3_) and succinic anhydride (C_4_H_4_O_3_) were obtained from Alfa Aesar. Chloroform (CHCl_3_), hexafluoro-isopropanol (C_3_HF_6_O) and methanol (CH_3_OH) from Sigma-Aldrich (New York, NY, USA) were used to solubilise and to precipitate the oligomers. All chemicals were used as received, without further purification.

Banana fibres were extracted from banana pseudo-stems collected from an industrial banana plantation (Plantations haut Penja, PHP (Njombé, Cameroon)) after the mature fruits had been harvested.

### 2.2. Glycolysis Reaction

**Figure 2:**
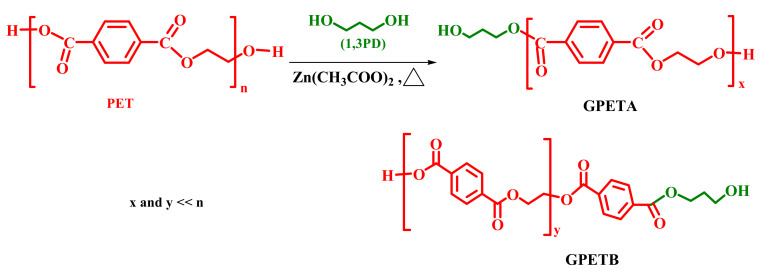
Reaction scheme of the PET glycolysis.

The glycolysis of PET was carried out in a 250 mL flat-bottom flask equipped with a reflux condenser and a magnetic stirrer. Propane-1,3-diol was used as a glycolysis reactant. In all experiments, 17.5 g (7.45 × 10^−4^ mol) of PET was mixed with 1.7 g (2.2 × 10^−2^ mol) of propane-1,3-diol and 0.1% wt of zinc acetate (based on total mass of PET and glycol). A 10 mL quantity of methanol was added to the slurry to homogenise the dispersion in the reactor. After evaporation of the methanol at 65 °C, the reaction mixture was heated to the depolymerisation temperature of 175 °C for 30 min followed by another heating at 200 °C for 120 min. The aim of the glycolysis was to obtain PET oligomers with at least 20 monomer units. After glycolysis, the reaction mixture was dispersed in 2000 mL of hot distilled water at 90 °C and filtered on a sintered glass crucible (porosity 3) under vacuum to remove the excess of unreacted glycol and the soluble fraction of glycolysed PET (co)-oligomer. The water-insoluble glycolysed PET (co)-oligomers were dried at 70 °C for 12 h. The reaction scheme is shown in Figure 2.

### 2.3. Co-Polyester Melt Condensation

**Figure 3:**
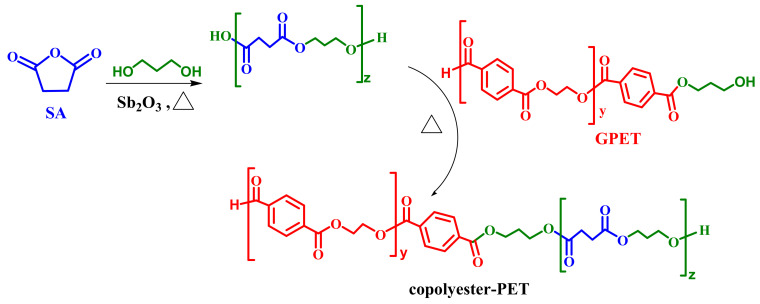
Reaction scheme of the co-polyester synthesis.

Co-polyesters were prepared in the melt from glycolysed PET, succinic anhydride (SA) and propane-1,3-diol (1,3-PD) under a nitrogen atmosphere. The reaction was performed via a two-step transesterification/polycondensation in a three-necked glass reactor equipped with a magnetic stirrer, a nitrogen inlet tube and a distillation column to condense water as well as low molecular mass reactants out of the reaction medium. The additional diol is unnecessary in theory but essential in practice to guarantee homogeneous reaction conditions. The initial overall COOH/-OH molar ratio was set at 1.01:1. An electronic probe was used to monitor the temperature of the reaction. Antimony oxide (Sb_2_O_3_) (0.1% wt based on total mass of glycolysed PET oligomer) was used as a catalyst for the reaction. SA and additional 1,3-PD in a molar ratio of 1:3 were first loaded into the reactor and the mixture was heated to 125 °C for 2 h to produce a predominantly acid-terminated oligomeric chain extender. The glycolysed PET was next added and the temperature raised to 205 °C for 20 h. After the reaction, 50 mL of chloroform was added to dissolve the co-polyester. The mixture was next transferred to a separating funnel and mixed dropwise with 750 mL of methanol to precipitate the co-polyester. Filter paper (porosity between 8–12 µm) was used to separate the two phases (liquid and solid). The solid phase was washed with distilled water and dried at 35 °C for 48 h under vacuum. The chemical reaction scheme for the synthesis of the co-polyester is described in Figure 3.

### 2.4. Molar Mass Build-Up by Soft Solid-State Polycondensation (S^3^P)

This reaction was performed on 0.5 mm-thick films of melt-polymerised samples supported on Teflon surfaces and in an evacuated oven (1 mbar) at temperatures ranging from 160 to 190 °C for up to 15 h. The films were cast from a chloroform solution and the solvent eliminated to constant weight. When heated to the reaction temperature in the oven, the films become rubbery soft. Here, their crystallinity is very low, but they allow a “soft solid state polycondensation” (S^3^P) provided the temperature program is chosen carefully. In some experiments, additional SA was mixed with the polymer solution to enhance the molecular weight build-up by esterification of the chain ends during S^3^P.

### 2.5. Banana Fibres Treatment

Banana fibres were manually extracted from the pseudo stem of banana plants (*Musa acuminata*). Then, the fibre bundles were mechanically separated into discrete technical fibres and treated with 0.125 N (5%) of sodium hydroxide at 70 °C for 1 h to dissolve hemicellulose and lignin [25,26]. The fibres were next washed with distilled water to neutral pH to avoid cellulose degradation during composite processing. The treated fibres were quenched in liquid nitrogen for 1 h before grinding in a variable speed rotor mill to obtain a fibre length comprised between 0.5 and 5 mm. Finally, the treated fibres were dried at room temperature under ventilation for 24 h.

### 2.6. Composites Processing

The chopped fibres were dried at 105 °C under vacuum for 12 h to remove moisture and to prevent polymer degradation by hydrolysis. The co-polyesters were also dried at 60 °C for 24 h for the same reason. Neat solid-stated co-polyester (COPET11) and the corresponding composites containing 5, 10 and 20% wt were compounded in a DSM conical twin-screw extruder in batch mode. The set temperature of the heating zone was 180 °C and 190 °C for the die. The screw speed was 100 rpm and the residence time in the extruder was about 2 min. The extruded composites were pelletised. The pellets were dried at 50 °C under vacuum for 24 h before injection moulding to produce dog-bone test pieces (ISO 527-2-5A) in a Thermo-Scientific HAAKE MiniJet Pro. The cylinder temperature was set at 190 °C, the mould temperature at 50 °C, the pressure at 600 bars and the post-pressure at 500 bars, all maintained for 30 s.

### 2.7. Characterisation Techniques

#### 2.7.1. Fourier Transform Infra-Red Spectroscopy (FTIR) Analyses

Hydroxyl and acid values of the starting PET, amorphous extruded PET, glycolysed PET and co-polyesters were determined by FTIR using a chain-end quantification method developed earlier [27,28]. FTIR analyses were recorded on a Nicolet Nexus 870 FTIR Infrared spectrometer. Films were produced by compression moulding of PET flakes at 250 °C for 30 s and 5 bars, then quenched in water to obtain an amorphous polymer and thoroughly dried in an oven at 70 °C under vacuum overnight to eliminate all traces of water (this is critical). Film thicknesses were calculated from absorbance at 1953 cm^−1^ and normalised to 200 µm for the FTIR analysis. Absorbance of the peaks at 3550 cm^−1^ and 3270 cm^−1^ were used to determine the concentrations of hydroxyl (C_OH_) and acid (C_COOH_), respectively, using the Lambert–Beer law Equation (1) [27,28]. The corresponding number-average molar mass (*M_n_*) of PET (co-polymers) was determined by Equation (2), reflecting the fact that all chains have a combination of acid and hydroxyl chain ends.

(1) $$C_{i}=\frac{A_{i}}{\in_{i}l}$$

(2) $$M_{n}=\frac{2}{C_{T}}$$

where $C_{T}=C_{OH}+C_{COOH}$ is the total concentration of acid and hydroxyl chain ends, *A_i_* the absorbance, *ε_i_* the molar absorptivity and *l* the path length.

#### 2.7.2. Intrinsic Viscosity (IV)

The intrinsic viscosity of the starting PET, glycolysed PET and PET co-polymers were determined with the help of an Ubbelhode viscometer at 30 °C in a mixture of hexafluoro- isopropanol (HFIP) and chloroform (CHCl_3_) (2:98 *v/v*) at room temperature [29]. Polymer concentration (*c*) was 0.05 mg/L in all cases. Usually, intrinsic viscosity (*IV*) is evaluated by extrapolation of the reduced viscosity at concentrations near zero. In this work, the Solomon equation was used [30]. This equation (Equation (3)) gives a result equivalent to the extrapolation of the reduced viscosity.

(3) IV=1c2tt0−lntt0−112

where *t*_0_ and *t* are the flow times of the pure solvent and of the solution respectively. For each sample, three measurements were made to improve accuracy and the average value was reported. The corresponding weight average molar mass (*M_w_*) was determined using the Mark–Houwink equation [31]:

(4) $$IV=1.91\times 10^{-4}M_{w}^{0.734}$$

#### 2.7.3. Differential Scanning Calorimetry (DSC) Analyses

A differential scanning calorimeter DSC1 from Mettler Toledo (Greifensee, Switzerland) was used to determine the glass transition temperature (*T_g_)*, the melting temperature (*T_m_*), the melting enthalpy (*ΔH_mi_*), the crystallinity (*X_i_*) and the crystallisation temperature (*T_c_*) of the samples. DSC analyses were carried out under nitrogen with sample masses of about 10 mg. The investigated temperature range of the first heating was from 30 to 280 °C at a rate of 10 K/min, followed by a cooling at 280 to 30 °C at −10 K/min and a second heating from 30 °C to 280 °C at 10 K/min. The melting temperature *T_m_* and crystallinity $X_{i}$ were determined from the first and second heating scans and the glass transition temperature only from the second heating [32]. As detailed in the Supplementary Section S1, the *T_m_–T_c_* relationship obtained for the co-polymers on an ultrafast DSC extrapolates to the same thermodynamic melting point as neat PET, which shows that the crystalline lattice of the co-polymers is identical to that of PET. Hence, the co-units are excluded from the crystalline phase. Crystalline fractions were thus calculated using the same equation as for PET:

(5) $$X_{i}=\frac{∆H_{mi}}{∆H_{m}^{0}}\times 100$$

where $∆H_{m}^{0}$ (=140 J/g) is the melting enthalpy of neat PET at 100% crystallinity [33].

The same protocol for crystallization and melting of the copolymer as used in the work of Nutenki et al. was followed, i.e., a two-stage annealing with a nucleation temperature of 75 °C and growth temperatures between 75 and 150 °C [34].

#### 2.7.4. Thermogravimetric Analysis (TGA)

Sample weight losses were analysed using a TGA/SDTA 851 from Mettler Toledo (Greifensee, Switzerland) and 8–10 mg samples. The samples were heated at 10 K/min under a nitrogen atmosphere.

#### 2.7.5. Dynamic Mechanical Analysis (DMA) and Tensile Tests

The dynamic mechanical properties of the composites were analysed in tensile mode using a DMA/SDTA861 testing machine from Mettler Toledo (Greifensee, Switzerland) equipped with a 40 N load cell. The samples were obtained by cutting dog-bone mouldings to average size (9 × 4 × 2.5 mm^3^), and measurements were performed on at least three samples for reproducibility. The heating scans were performed at a constant frequency of 1 Hz, a 3 K/min scan rate from 20 to 150 °C, a maximum force of 2 N and a maximum displacement of 1 μm.

Tensile tests were carried out on as-moulded co-polymer samples after SSP and their composites. The samples were tested at a relative humidity of 50% at room temperature using a Zwick Roel test machine (Ulm, Germany) at a crosshead speed of 5 mm/min. These tests were performed on 5 specimens for each batch.

#### 2.7.6. Scanning Electron Microscopy (SEM)

SEM analyses were performed on banana fibres (untreated and treated) and on fracture surfaces of tensile test specimens using a JEOL 7600F instrument (JEOL, Ltd., Tokyo, Japan) operated at 1.5 kV. The surfaces of the fibres and composite fracture surfaces were coated with platinum by sputtering to avoid electrical charging.

## 3. Results and Discussion

The sequence of polymer and fibre modifications followed by composite preparation is described in Figure 1 to help the reader picture it.

### 3.1. PET Glycolysis

**Figure 4:**
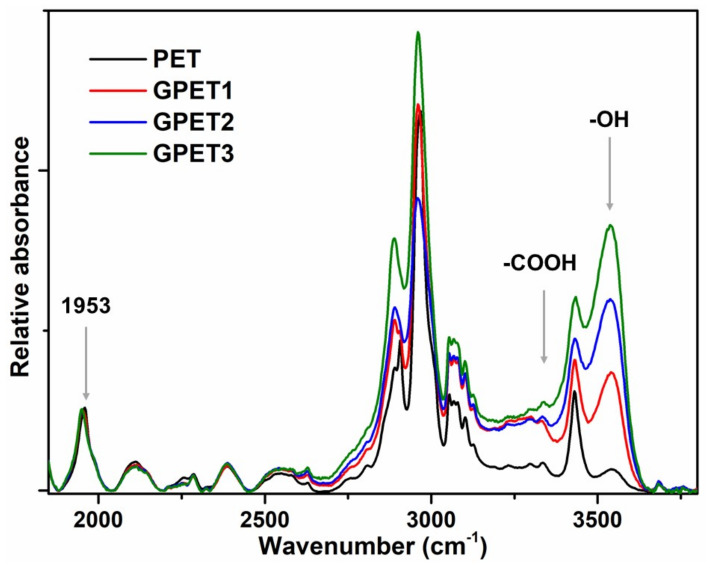
FTIR analysis of extruded and glycolysed PET.

Three different molar ratios of 1,3-PD with respect to PET units were used for the glycolysis (10, 20 and 30). The resulting oligomers were characterised by FTIR for the quantification of the chain ends and DSC for the thermal properties. The details of the reaction mixtures and characterisation results are presented in Table 1. The FTIR spectra of the commercial and glycolysed PET (GPET1, GPET2 and GPET3) are shown in Figure 4 after normalisation of the film thickness to 200 μm [27,28]. The intensity of certain absorption bands increased for the glycolysed oligomers compared with the starting PET, which can be related to the increased concentration of OH and COOH end groups. Hydroxyl chain-ends increased dramatically whereas carboxylic ones increased only moderately. The hydroxyl concentration increases due to the statistical chain clipping of the polymer ester groups by the diol (transesterification). The increase in the acid-end-group concentration is less obvious to explain. It is certainly not due to beta scission, which is only active at much higher temperatures [35,36,37]. The most likely explanation is the significant difference between the partition coefficients in hot water of the acid and hydroxyl terminated oligomers of very low molar mass. In particular, terephthalic acid is insoluble in water whereas propane diol is fully miscible. Hence, the acid end groups concentrate more in the water-insoluble fraction via a purely physical effect. This also means that a quantitative assessment of the glycolysis efficiency measured using FTIR analysis of the sole insoluble fraction is rather inaccurate (Table 1). The vibration bands at around 2870 and 2950 cm^−1^ result from the asymmetric and symmetric stretching of the C–H bonds of methylene groups. Higher amounts of diol-to-PET ratios logically reduce the final molar mass and the melting point of the oligomers. Concurrently, the crystallinity of the reaction products increases. GPET3 (Table 1) was selected for the next steps.

The molar concentration of 1,3-PD in GPET3 and its molar fraction with respect to the repeat units can be calculated from the increase in the –OH end-chain concentration caused by glycolysis. The molar concentration is half the hydroxyl value increase. It does not matter if the diol reacted once or twice. Neither is the calculation affected by the partition of the low molar mass oligomers between the soluble and insoluble fractions since only the insoluble fraction of the oligomer is of interest and the starting PET is fully insoluble in water. The result is 0.2345 meq/g from Table 1. This value is small enough compared with the bulk molar concentration of PET repeating units, i.e., 5.21 meq/g, to allow uncorrected calculation of the 1,3-PD molar fraction in GPET3, which comes up to approximately 4.5%. This means that there are about 20 unperturbed PET units on average between modified ones. The chain extension by succinic anhydride detailed in the following section potentially alters this picture (discussion in Section 3.2).

**Figure 5:**
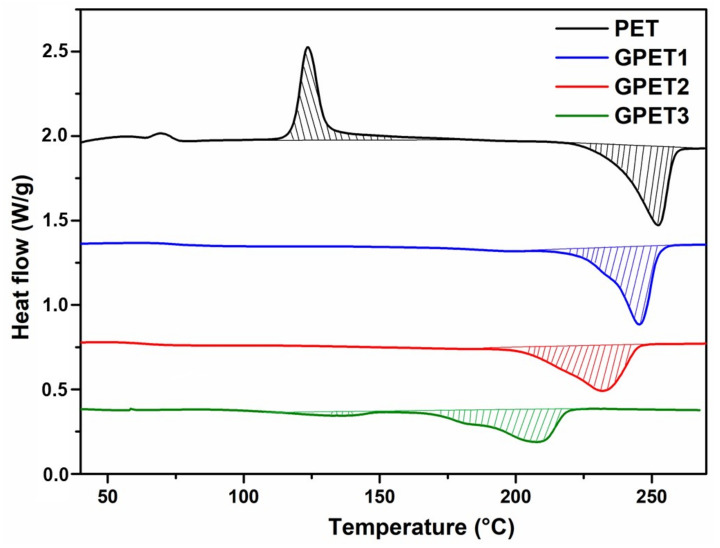
DSC first heating scans of extruded PET and glycolysed PET oligomers.

Figure 5 shows the melting peaks (first heating scan) of the starting PET compared with the glycolyzed oligomers. A higher 1,3-PD concentration leads to a lower melting point because the concentration of chain ends acting as defects increases and the molar mass decreases by the same proportion.

### 3.2. Melt Reaction of Glycolysed PET (GPET3)

PET co-polyesters were prepared by reacting the glycolysed PET oligomer GPET3 in two steps as described in Section 2.3. The molar mass of the resulting co-polymer mainly increased by esterification of the carboxyl end chains of the chain extender (pre-reacted 1,3-PD with succinic anhydride) with hydroxyl chain ends of the glycolysed PET involving the elimination of water molecules under nitrogen purge. Various stoichiometric ratios were studied, and the key results are summarised in Table 2. The co-polymers were characterised by FTIR and DSC to evaluate molar masses as well as melting and glass transition temperatures. The evolution of the reaction was mainly affected by the fraction of succinic anhydride (SA). Increasing the concentration of aliphatic ester units reduced the melting and glass transition temperatures of the resulting co-polyesters [38]. This can be observed in Table 2 by comparing COPET1 and COPET4 (high and low aliphatic ester units, respectively). On the other hand, increasing the amount of SA while leaving the other ratios constant mainly reduced the final molar mass (compare COPET2 and COPET3).

Assuming full conversion during the chain-extender synthesis step and no loss of reactants, it is possible to estimate the number average degree of polymerisation of the chain extender from the diol/SA ratio by using the Carothers equation. In the case of COPET4, the ratio *r* of functional groups is 2/3 and the resulting degree of polymerisation is (1 + *r*)/(1 – *r*) = 3. Very roughly, since quantitative accuracy is impossible due to the many assumptions, the molar concentrations of oligomer and chain extender were comparable for this composition; hence, the unperturbed PET unit sequence length of about 20 was not dramatically reduced unless the chain extended oligomers trans esterified extensively during the subsequent melt polycondensation.

**Figure 6:**
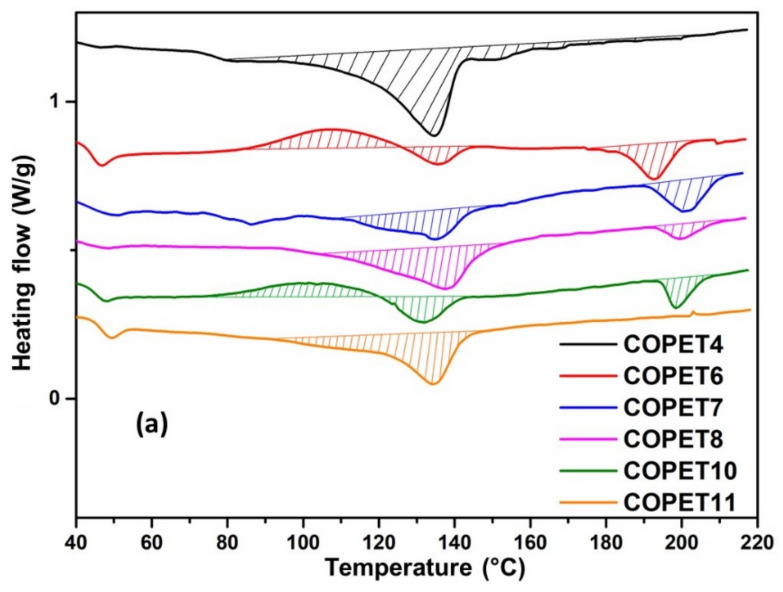

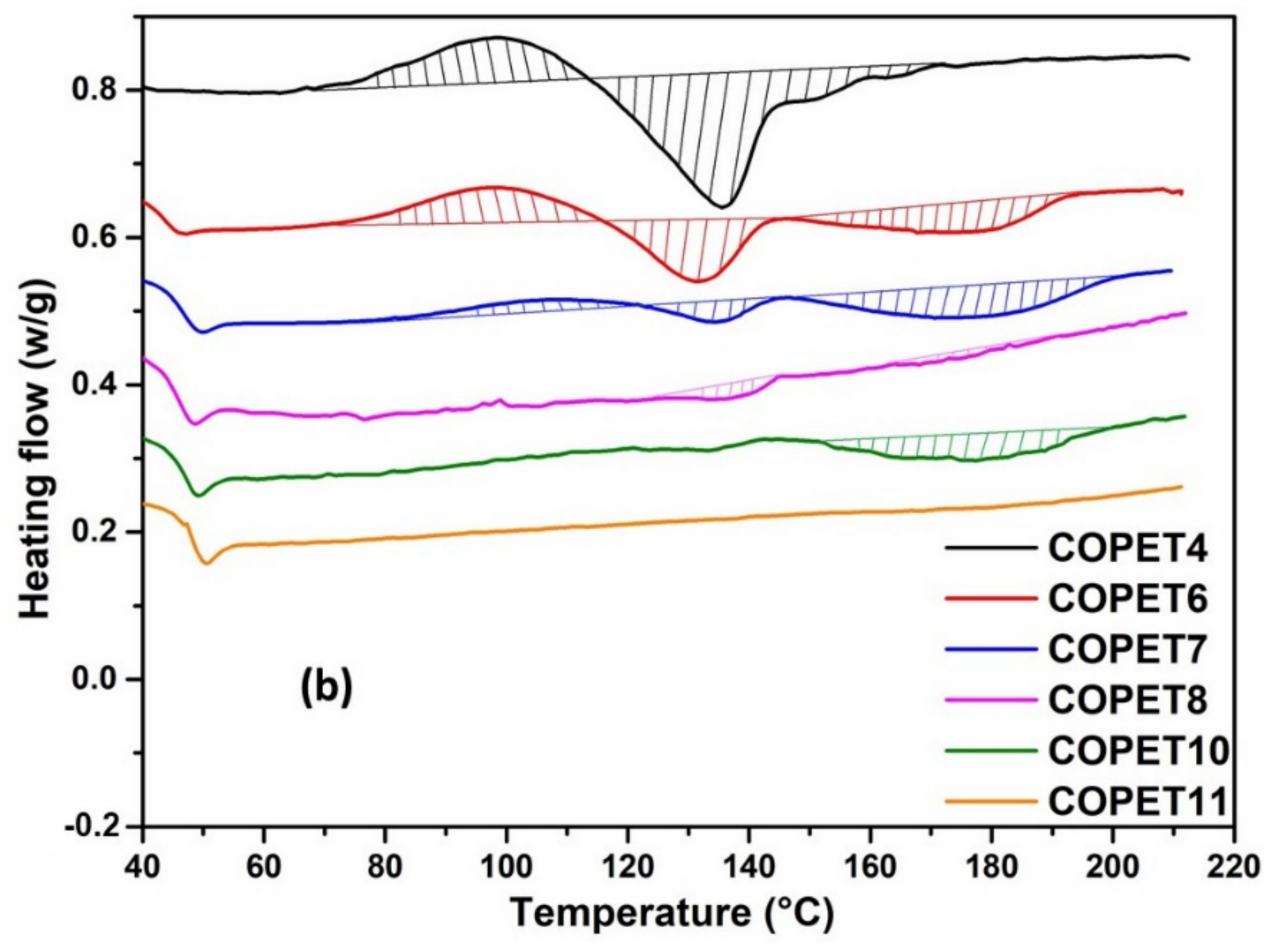
Influence of SSP temperature on thermal properties measured by DSC: (**a**) first heating and (**b**) second heating.

As shown in Figure 6a, the sole peak melting temperature of COPET4 (cast film) is not sufficient to correctly describe its melting behaviour since the melting peak is quite broad and has a shoulder extending up to about 160 °C. This feature is of significance for the solid-state polymerisation experiments discussed in the next paragraph.

COPET4 was selected as the starting material for the SSP because of its acceptable *T_g_*. However, its molar mass is insufficient for suitable mechanical properties.

### 3.3. S^3^P and SSP of Melt Polymerised Co-Polyester COPET4

Solid-state polymerisation (SSP) is an effective way to increase the molar mass of semi-crystalline PET at temperatures between *T_g_* and *T_m_* in a vacuum or under inert gas flow; thus greatly reducing the high activation energy of side-reactions that limit the molar mass build-up in melt polycondensation. Moreover, as the activation energy of esterification is lower than that of transesterification, the former is favoured to the extent that the reaction temperature is lowered [22]. SSP only takes place in the interlamellar amorphous zones of the semi-crystalline polymer.

In a first series of experiments, SSP was performed on thin films of COPET4 cast from a chloroform solution without further modification (details in Section 2.4). The reaction program involved isothermal steps of 2 h at temperatures ranging from 160 °C to 190 °C with increments of 10 °C. The justification of this careful progression was to retain a minimal solid character for the films during the whole process, which was achieved up to 190 °C (COPET8) as shown in Figure 6a.

Table 3 summarises the SSP conditions and corresponding characterisation results, including the evolution of the chain-end concentrations, the intrinsic viscosity in CHCl_3_-HFIP and the corresponding weight average molar mass. The higher the final reaction temperature up to 190 °C, the higher the molar mass, which is due to the higher segmental mobility and reactivity at higher temperatures in the amorphous phase of the semi-crystalline films. The SSP temperature completely dominates the molar mass build-up. Noticeably, the more reactive –COOH end groups decrease much faster than the –OH ones.

The thermal characterisation of the films is presented in Figure 6 (first heating scan (a) and second heating scan (b)) and the key results are tabulated in Table 3.

**Figure 7:**
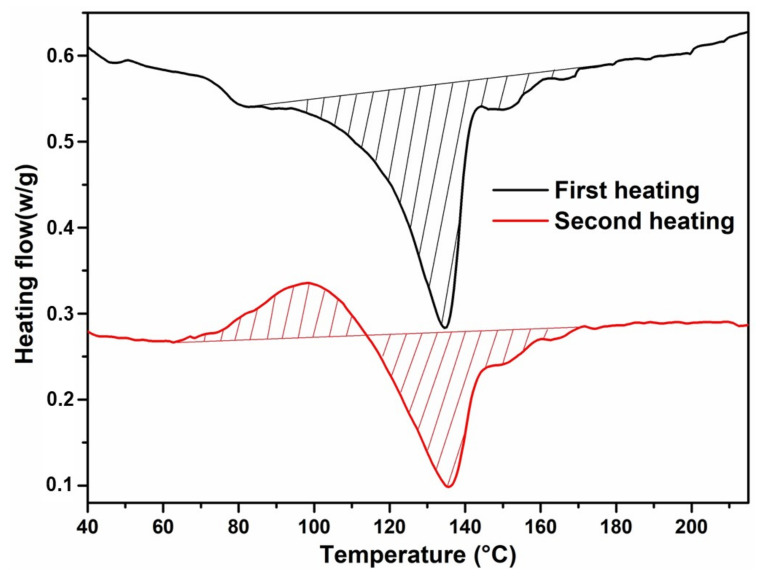
DSC analysis of COPET4 film heating range 25/220 °C.

After complete elimination of the solvent, the melt-polymerised COPET4 films cast from chloroform were semi-crystalline with a crystallinity of about 24% (based on the PET pure crystal melting enthalpy of 140 J/g) [33] and melt over a broad temperature range from about 100 to 160 °C with a peak at 135 °C (Figure 6a). This low melting range is explained by the quickly decreasing polymer segmental mobility when the solvent evaporates, preventing the growth of thick lamellae. If COPET4 films were heated to a maximum temperature of 220 °C in the DSC, the samples remained crystalline after cooling and the melting temperature range was restored without change (Figure 7). In the 160 °C to 190 °C range, the films felt rubbery, were slightly sticky and could be (carefully) manipulated as a soft solid. Clearly, above the melting range and up to at least 220 °C, a tiny fraction of physical bridges survived, presumably as self-seeded crystalline nuclei, maintaining some mechanical integrity in the films and permitting a modified version of solid-state polymerisation (SSP) in a quasi-molten state. This process was coined “soft solid-state polycondensation” S^3^P. This type of polycondensation has not been extensively described in the literature. It has the advantage that the reactive domain covers essentially 100% of the volume compared with the usual SSP. On the other hand, if a COPET4 film is heated up to 280 °C, it remains amorphous upon cooling and even when reheated (not shown). After 2 h at 160 °C followed by 2 h at 170 °C (COPET6, Figure 6a), the films became truly crystalline with a peak melting temperature of 193 °C and a low crystalline fraction of about 6%. Hence, the next stages were classical SSP rather than S^3^P. The films reacted at higher temperatures (COPET7 and COPET8 at 180 and 190 °C, respectively) and showed even higher melting peak temperatures but decreasing, albeit non-zero, crystallinity (Table 3). The cold crystallisation peaks around 100 °C and secondary low melting peaks at around 135 °C observed on all films were due to the cooling of the films when taken out of the oven, followed by reheating during DSC analysis and are not representative of the SSP as such.

*T_g_* remained unchanged at 48 °C during the set of isothermal reactions. On the other hand, the crystallisation kinetics were significantly affected as the molar mass increased. The second heating scan (Figure 6b) shows that the co-polymers reacted at the highest temperatures and crystallised too slowly to develop significant crystallinity upon cooling at 10 °C/min.

Table 3 shows a large imbalance between a low –COOH concentration and a large –OH concentration in all the films that have undergone S3P/SSP (COPET6 to 9). This is clearly detrimental to the molar-mass-build-up kinetics when the main reaction is esterification. The addition of small amounts of SA to COPET6 (chosen because it shows this end-group discrepancy), followed by SSP, was tested to check if this route can indeed boost the molar mass build-up. To this end, COPET6 samples were mixed with small amounts of SA (0.5 and 1 *w*/*w*%) in chloroform at room temperature and the solvent evaporated to constant mass to produce films as described in Section 2.4. The films were reacted at 170 °C under the same SSP conditions as above for 15 h.

The characterisation results are summarised in Table 4 and Figure 6. Table 4 shows the clear benefits of adding SA at the 1% level. SA presumably first reacts with co-polymer –OH chain ends, producing acid chain ends without molar mass reduction, and those chain ends further react with more OH chain ends, favouring additional molar mass build-up by direct esterification. Moreover, the additional SA probably also increases mobility in the amorphous phase. Table 4 also shows that 1% SA does indeed reduce the –OH/–COOH imbalance of the starting co-polymer.

Figure 6 shows the first and second DSC heating scans of the co-polymers with SA added after SSP/S^3^P (COPET10 and COPET11). COPET10 (0.5% SA) shows a melting peak around 200 °C during the first scan and a melting peak around 180 °C during the second. It was therefore semi-crystalline during the reaction at 170 °C and underwent a classical SSP. The melting peak around 135 °C can be explained, as above, as resulting from recrystallisation of the sample when taken out of the oven. On the other hand, COPET11 (1% SA) only shows a low melting peak around 135 °C during the first scan (which is not present at 170 °C of course) and no melting peak during the second scan. It behaves, therefore, like COPET4, i.e., no detectable crystallinity at 170 °C but a remaining “ghost network” of crystalline nuclei and rather undergoes S^3^P, which still leads to successful molar mass build-up and good mechanical properties as shown below.

### 3.4. Banana Fibre Treatment and Morphology

The chemical functions before and after alkaline treatment were investigated by FTIR spectroscopy. The results detailed in the Supplementary Section S2 confirm that the treatment removed a significant amount of low stability components such as pectins and lignin as well as partially removing hemicelluloses. The treatment further decreased the formation of intramolecular hydrogen bonds due to ionisation of pectin, hence favouring the dispersion of the fibres in the matrix. The thermal stability of the fibres before and after treatment was analysed using TGA. The results are detailed in the same Supplementary Section S2. The treatment of the fibres increased the thermal stability by increasing the temperature of the main degradation peak by 50 °C, from ~300 °C to ~350 °C, which is assigned to the removal of pectineus substances and the corresponding increase in the cellulosic content. The fibres will only be exposed to 170–200 °C during the melt processing of the co-polyesters (composites) developed in this work, whereas temperatures as high as 280 °C are unavoidable in the case of neat PET.

**Figure 8:**
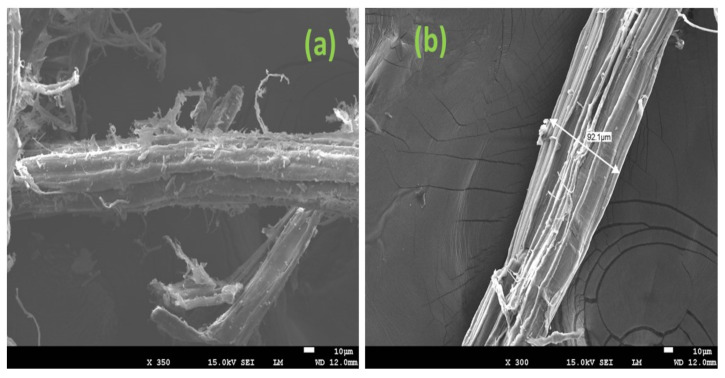
SEM images showing the influence of treatment on banana fibre morphology: untreated (**a**) and treated (**b**) banana fibres.

SEM analyses were performed on treated and untreated banana fibres to better understand the influence of the treatment on the morphology. Figure 8a (untreated fibres) shows the usual structure with a rough texture consisting of cellulose, hemicelluloses, lignin, pectins and waxes, the latter component giving natural resistance to the banana pseudo-stem. After alkaline treatment (Figure 8b), a moderate reduction in fibre diameter is observed and some structural components are removed. In particular, the treated banana fibres lose the attached fibrils and deposits characteristic of the untreated fibres.

### 3.5. Tensile Properties of Co-Polyesters Compared with PET

**Figure 9:**
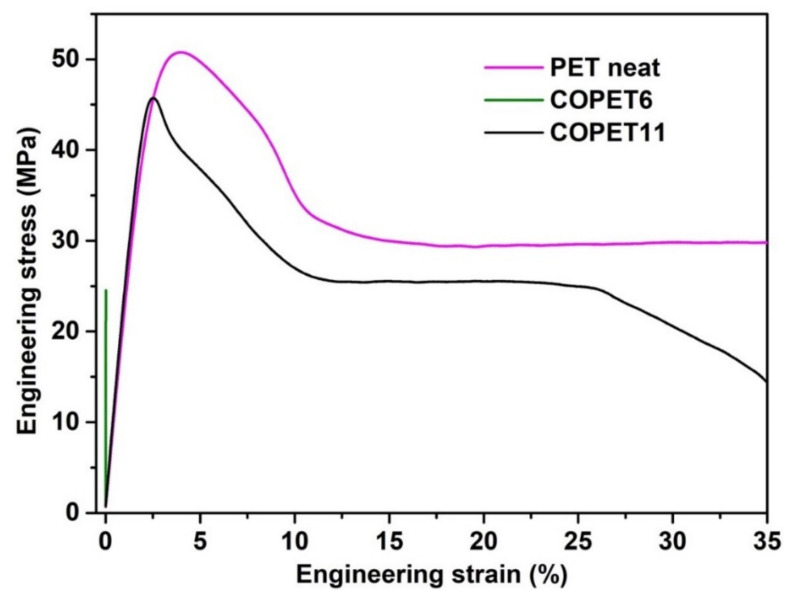
Tensile test of neat PET, COPET6 (SSP without added SA) and COPET11 (SSP with 1% added SA).

The tensile properties of neat PET and two co-polyesters after SSP, i.e., COPET6 without added SA and COPET11 with 1% SA (see Table 4), are presented in Figure 9. COPET6 shows a very brittle, purely elastic behaviour with high elastic stiffness around 4500 MPa. This high modulus is the result of a high degree of crystallinity. However, the tensile strength of 25 MPa and the fracture strain of 0.1% are very low. The molar mass is clearly insufficient to yield adequate ductility. On the contrary, the tensile properties of COPET11 at RT are similar to those of commercial PET, with an elastic zone, a softening zone and a plateau with plastic deformation spreading progressively along the entire specimen. The Young’s moduli of the two materials were similar, at around 2500 MPa. The maximum elongation at break and yield stress of COPET11 were 45% and 45 MPa, respectively, close to those of neat PET. These results clearly demonstrate the advantage of balancing the -OH and –COOH end groups during SSP, hence favouring esterification rather than only transesterification. Interestingly, the high ductility of COPET11 was achieved at a lower molar mass than that of neat PET (see Table 4). This is due to the higher flexibility of the aliphatic co-units.

### 3.6. Properties and Morphology of the Composites

Because of its excellent mechanical properties, COPET11 was selected for the processing and characterisation of the composites (see Section 2 for details).

**Figure 10:**
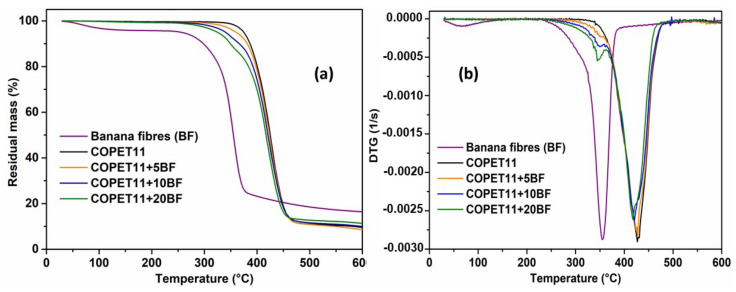
Thermal degradation of treated banana fibres, COPET11 and composites with different fibre contents, (**a**) TGA and (**b**) DTG.

The thermal stability of COPET11 and of composites with 5, 10 and 20% wt treated fibres are shown in Figure 10a,b. The TGA and DTG curves of treated fibres are shown for reference. The composites exhibit two degradation zones. A first weight loss due to the fibres occurs at around 350 °C. It ranges from 3 to 15% depending on fibre content from 5 to 20%. Therefore, the weight loss curve of the composites suggests a slight protection of the fibres by the matrix. The temperature of maximum degradation rate of the neat matrix and composites occurs around 425 °C. When the amount of fibre increases, this temperature decreases slowly. The final mass loss of the matrix and composites is around 85%, surprisingly little affected by the fibre content.

**Figure 11:**
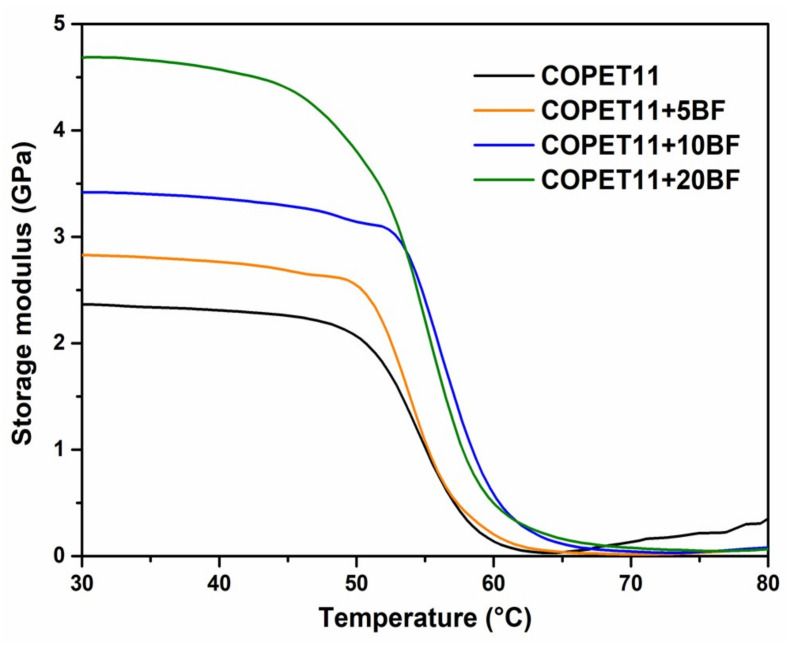
Storage moduli of COPET11 and composites.

Dynamic mechanical thermal analyses (DMTA) and tensile tests were performed on the neat matrix and composite samples of up to 20% banana fibres after the extrusion and injection moulding processes without further thermal annealing treatment. The effect of the banana fibre concentration on the dynamic storage modulus of COPET11-BF composites is compared to neat COPET11 in Figure 11. The only observed transition is *T_g_* around 60 °C for all samples (from tan delta maxima, not shown), consistent with the DSC results reported above considering the difference between the two methods. The independence of *T_g_* from fibre content suggests the chain mobility reduction by the matrix–fibre interfaces is insignificant. Moreover, the matrix clearly remains amorphous because the crystallisation kinetics are too slow, which is consistent with DSC results on the neat matrix. However, the DMTA further shows that there is no major nucleation effect of the fibres within the processing window explored.

The storage modulus of neat COPET11 is equal to 2.3 GPa at 25 °C, close to neat PET. Increasing fibre content in COPET11 up to 20% wt raised the storage modulus from 2.3 to 4.6 GPa. Beyond the glass transition, the modulus falls abruptly as the material changes from glassy to rubbery. Although the observation is not very precise, the storage modulus tends to increase again above *T_g_*. The possible explanations for this include a recrystallization of the matrix and a consolidation at the fibre–matrix interface.

**Figure 12:**
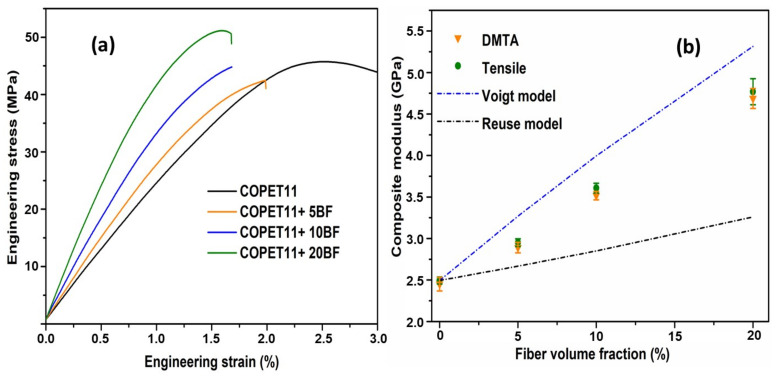
Tensile properties of neat COPET11 and banana fibre composites at 25 °C as a function of the fibre fraction. (**a**) Experimental stress-strain curves, (**b**) experimental and Young’s modulus vs. fibre content, compared with Voigt and Reuss bounds.

The results of tensile tests performed at 25 °C are reported in Figure 12 as a function of fibre content. They show an increase in Young’s modulus and a corresponding decrease in the elongation at break with increasing fibre content in the COPET11 matrix. The 20% composite still shows small amounts of plasticity but the 5 and 10% composites show a brittle fracture.

Figure 12b compares the measured Young’s moduli with the theoretical lower and upper bounds (Reuss and Voigt models) for the elastic modulus of composites as expressed by the following equations [34]:

(6) $$\mathrm{Reuss}\;\mathrm{model}:\;\frac{1}{E_{c}}=\frac{\emptyset_{m}}{E_{m}}+\frac{\emptyset_{f}}{E_{f}}$$

(7) $$\mathrm{Voigt}\;\mathrm{model}:\;E_{c}=E_{f}\emptyset_{f}+E_{m}\emptyset_{m}$$

where *E_c_*, *E_m_* and *E_f_* are the moduli of composite, neat matrix (COPET11) and banana fibres, respectively. $\emptyset_{m\;}$ and $\emptyset_{f}\;$ are the volume fractions of the matrix and fibres, respectively.

The application of Voigt and Reuss bounds assumes no porosity or other significant internal flaws [39]. This was indeed verified. The fibre weight fractions must be converted to volume fractions (∅) from the density of fibres and matrix. The density of the co-polymer matrix was assumed to be close to that of neat PET, which is 1.3 and is the same as that of banana fibres [20,40]. The Young’s modulus of single banana fibres is around of 30 GPa [41,42]. Technical banana fibres with diameters of 70–100 μm were used in this work, whose Young’s modulus is lower, at around 17 GPa [23]. The modulus of COPET11 (*E_m_*) was taken at 2.5 GPa from the tensile tests.

The tensile modulus of the composites increased from 2.5 GPa to 4.8 GPa when the fibre fraction (identical mass and volume fractions assumed) increased from 5 to 20%. The experimental moduli of the composites were comprised between the two theoretical bounds but closer to the Voigt upper bound, which means that the banana fibres carry the maximum load possible as a result of a full transfer of the applied stress [34].

**Figure 13:**
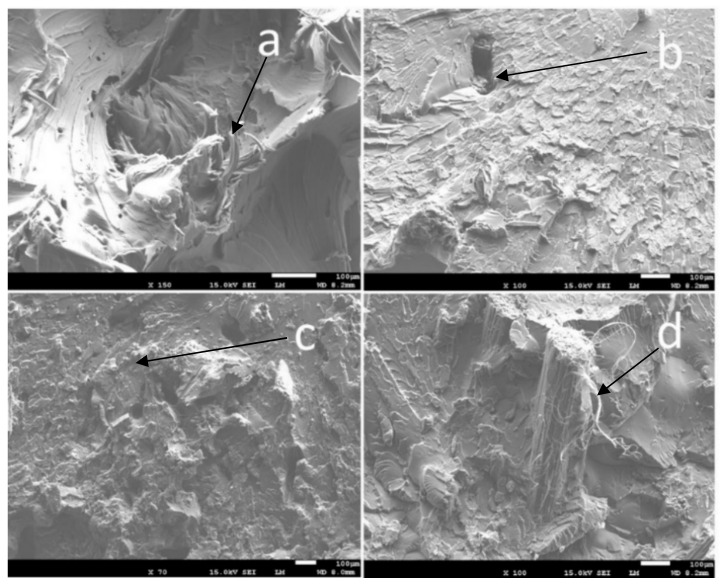
SEM of fracture surfaces: neat COPET11 (**a**), COPET11 + 5% BF (**b**), COPET2 + 10% BF (**c**), and COPET11 + 20% BF (**d**).

The fracture surfaces from broken dog-bone tensile specimens of neat COPET11 and composites with different fibre contents (5 to 20% wt) were characterised by SEM with a focus on the interface between the fibres and the matrix (Figure 13). Neat COPET11 presents a very rough cross section and fibrillar deformation, characteristic of the plastic deformation of a ductile polymer. In the composite materials, the fibres are well dispersed and show excellent adhesion with the matrix. Fibres are mostly oriented parallel to the length of the specimens, i.e., perpendicular to the fracture surface. This is related to the high shear experienced by the matrix and fibres during the moulding process and likely explains why the stiffness is closer to the Voigt upper bound than the Reuss lower bound (see Figure 12). The very clean breaks for the fibres in the matrix and evidence of fibrillation at the fibres surface (e.g., Figure 13d, black arrow) indicate a high interfacial strength that suggests covalent bonding by (trans)esterification between the fibres and the matrix.

### 3.7. Elementary Sustainability Analysis of the COPET+BF Composites

As a first attempt towards quantifying the benefits of our approach to recycled PET composites in terms of a property–sustainability balance, the material selection method proposed by M. F. Ashby [43] was used to construct a material index for the combination of stiffness and embodied energy. This performance index reflects the choice of the best material for a stiff beam with prescribed bending stiffness (constraint) and minimum embodied energy (objective). The relevant material index *M,* for an objective to minimise environmental impact is as follows [44]:

$$M=\frac{\rho H_{p}}{E^{1/2}}$$

where ρ is the density, *H_p_* the embodied energy per unit mass and *E* the Young’s modulus. Embodied energy represents the fossil fuel energy consumed to make one kilogram of material (including extraction, manufacture and transport) and it generally lies in the range of 50 to 250 MJ/kg for polymers. This value is reduced for recycled materials [45]. Embodied energy of a recycled material (10–100 MJ/kg) is lower because the intrinsic energy of first production is not included in this case, which is a valid assumption if many recycling steps are envisaged [44]. The embodied energies of banana fibres, recycled PET and a recycled PET composite with 20% wt BF were 2, 27 and 22 MJ/kg respectively.

**Figure 14:**
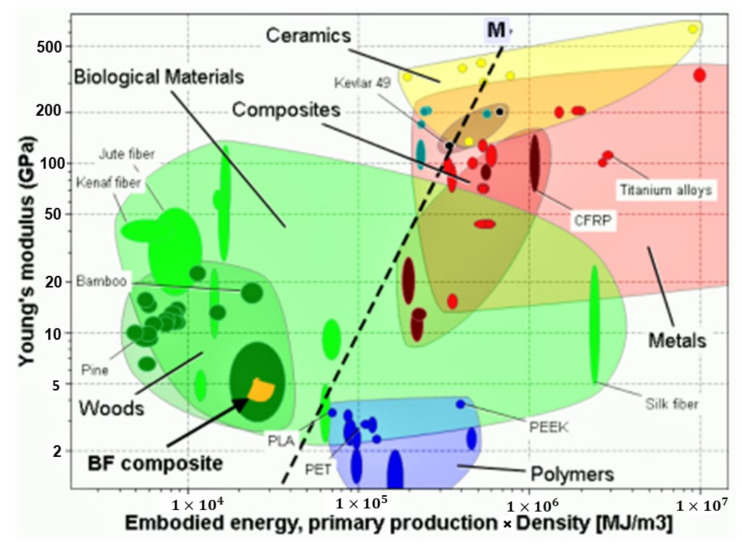
Selection chart for stiffness vs. embodied energy after screening of irrelevant materials. The superior materials are in the top left corner (high modulus for low embodied energy).

The composites developed in this work were added to the relevant materials property map of Figure 14, giving the Young’s modulus as a function of embodied energy. The chart suggests that the COPET+BF composites are very favourably positioned close to wood materials. The modulus of a composite with 20% banana fibres was 4.8 GPa and the corresponding embodied energy was around 30,000 MJ/m^3^. These values are indeed close to low-end wood materials. Moreover, one must of course remember the much larger versatility of the present material in terms of shaping and moulding compared with wood.

## 4. Conclusions

The motivation for this work was to explore the feasibility of upcycling used PET bottles, which represent a major environmental issue, especially in Equatorial and West Africa, by combining it with an abundant but neglected local natural resource, i.e., the fibres extracted from the banana plant. These fibres have attractive reinforcing properties, especially after a simple alkaline treatment (0.125 N NaOH at 70 °C for 1 h) that increases their thermal stability by 50 °C (weight loss peak by TGA). The major technical difficulty was the limited thermal stability of the fibres, preventing straightforward melt processing of the composites with unmodified PET, which requires melt processing temperatures of at least 280 °C. An integrated approach “from polymer chemical recycling to composite testing” was developed involving partial PET glycolysis followed by chain extension in the melt and a “soft” solid state to produce a crystallisable co-polyester with a *T_g_* of 48 °C and RT tensile properties close to the starting PET but with a processing temperature of 190 °C that is compatible with the thermal stability of the fibres. The main reactants used (1,3-propane diol and succinic anhydride) can be bio-sourced. The partial, as opposed to full, glycolysis preserved the main part of the embodied energy of the starting PET material.

The molar mass build-up of the co-polyester in a solid state occurring at unusually low temperatures (160° to 190 °C range) was dominated by esterification and was enhanced by a balanced ratio between acid and hydroxyl end groups. Moreover, some solid-state reaction steps took place in a quasi-molten state, which may be better qualified as “soft solid-state polymerisation”.

The composites obtained from the co-polymers showed an attractive balance of properties in terms of stiffness (4.8 GPa at 20% fibres), acceptable ductility (clear presence of a yield point) and embodied energy (30,000 MJ/m^3^) when discussed in the context of an Ashby-type analysis, bringing them close to low-end wood-based materials in terms of stiffness/embodied energy balance and confirming the environmental friendliness of the overall approach.

This work was limited to a laboratory-scale scientific and technical proof of concept and needs to be extended to assess the cost–properties balance at an industrial scale. Molar-mass-build-up kinetics and workup procedures need to be optimised. A further limitation of the co-polyester obtained from recycled PET was its slow crystallisation kinetics, which could be alleviated by nucleating the matrix.

The potential impact of the present work goes well beyond the PET–banana fibre composites studied, since the PET modification developed here can be used to prepare composites with different natural reinforcing fibres with comparable thermal stability and surface characteristics.

## Figures and Tables

**Table 1:** Glycolysis parameters and corresponding FTIR and DSC analyses.

Co-PET Oligomer	Molar Ratio Co-Diol/PET	Approx. Reacted Glycol (%)	Analysis of Glycolysed Co-PET			DSC Analysis		
			Acid Value (µeq/g)	Hydroxyl Value (µeq/g)	*M_n_* (g/mol)	*T_g_* (°C)	Peak *T_m_* (°C)	*χ* (%)
PET	-	-	42	43	23,500	76	250	78.0
GPET1	10	40.8	120	225	5800	76	245	33.0
GPET2	20	32.2	114	430	3700	76	231	30.0
GPET3	30	15	125	512	3125	76	207	25.0

**Table 2:** Influence of SA and 1,3-PD content on co-polymer molar mass build-up and thermal properties.

Co-Polyester Acronym	Glycolysed PET: Diol: Succinic Anhydride	FTIR			DSC Analysis Second Heating		
		Hydroxyl Value (µeq/g)	Acid Value (µeq/g)	*M_n_* PET (g/mol)	*T_g_* (°C)	Peak *T_m_* (°C)	*χ* (%)
COPET1	1:6:7	77	84	12,400	28	161	13.0
COPET2	1:6:8	205	52	7800	25	186	12.0
COPET3	1:6:10	362	64	4700	22	157	16.0
COPET4 precipitated	1:2:3	212	165	5300	48	135	10.0
COPET4 cast film	“	“	“	“	48	135	24.0

**Table 3:** Influence of temperature program on S^3^P/SSP reaction.

Sample	T SSP (°C)	Intrinsic Viscosity (dL/g) *	MW from IV (g/mol)	Chain Ends from FTIR			DSC Analysis First Heating			
				[COOH] (μeq/g)	[OH] (μeq/g)	*M_n_* (g/mol)	*T_g_* (°C)	*T_m_*_1_ (°C)	*T_m_*_2_ (°C)	*χ_m_*_2_ (%)
COPET4-FILM	-	0.243	17,600	165	212	5300	48	134	152 (shoulder)	24
COPET5	2 h @ 160	0.252	18,500	158	124	7100	48	-	182.5	17.0
COPET6	+2 h @ 170	0.265	19,900	29	155	10,300	48	134	193	6.0
COPET7	+2 h @ 180	0.314	25,000	25	168	10,400	48	134	202	7.0
COPET8	+2 h @ 190	0.327	26,500	18	112	15,400	48	134	200	2.0

* in CHCl_3_-HFIP. COPET4 is soluble in pure CHCl_3_ but the solid-state samples (COPET5 to 8) are only soluble in the mixture.

**Table 4:** Effect of additional SA on SSP of COPET6.

Sample	T SSP (°C)	SA Additional Amount (% wt)	Reduced Viscosity (dL/g)	MW from IV (g/mol)	Chain Ends (FTIR)		DSC Analysis First Heating			
					[COOH] (μeq/g)	[OH] (μeq/g)	*T_g_* (°C)	*T_m_*_1_ (°C)	*T_m_*_2_ (°C)	*χ_m_*_2_ *(%)*
PET			0.53	49,700	42	43	78	250		8.0
COPET6	170	-	0.265	19,900	29	155	48	134	193	6.0
COPET6_AS1		0.5	0.265	19,900	-	-	-	134	197	7.0
COPET6_AS2		1	0.265	19,900	-	-	-	134	193	5.0
COPET10	170	0.5	0,293	22,800	22	212	48	134	198	3.0
COPET11	170	1	0,350	29,100	40	119	48	134	-	-

## Data Availability

The data presented in this study are available on request from the corresponding author.

## Supplementary Materials

The following supporting information can be downloaded at: https://www.mdpi.com/article/10.3390/polym14224791/s1, Figure S1: Melting enthalpy (a) and temperature (b) of the crystalline phase as a function of the crystal growth temperature for COPET4; Figure S2: Influence of banana fibres alkaline treatment on FTIR spectra; Figure S3: Influence of banana fibres treatment on thermal stability of banana fibers (a) TGA and (b) DTG. References [46,47,48,49,50,51,52] are cited in the “supplementary materials”.
