# Supplementary material for: Integrated Approach to Eco-Friendly Thermoplastic Composites Based on Chemically Recycled PET Co-Polymers Reinforced with Treated Banana Fibres
Source: Polymers (Basel). 2022 Nov 8;14(22):4791. doi: 10.3390/polym14224791 (PMC9693550; doi:10.3390/polym14224791)
Supplement: Supplementary file 1 [file polymers-14-04791-s001.zip › polymers-1984420-supplementary.pdf]

Supplementary Materials:

Integrated Approach to Eco-Friendly Thermoplastic Composites Based on Chemically Recycled PET Co-Polymers Reinforced with Treated Banana Fibres

Martial Aime Kuete [1,2,3], Pascal Van Velthem [1], Wael Ballout [1], Bernard Nysten [1], Jacques Devaux [1], Maurice Kor Ndikontar [2], Thomas Pardoen [3] and Christian Bailly [1,*]

[1] Institute of Condensed Matter and Nanosciences—Bio &Soft Matter (IMCN/BSMA),UCLouvain, 1348 Louvain-la-Neuve, Belgium
[2] Macromolecular Chemistry Unit, Applied Chemistry Laboratory, Faculty of Science, University of Yaoundé I, P.O. Box 812 Yaoundé, Cameroon
[3] Institute of Mechanics, Materials and Civil Engineering, UCLouvain, 2 Place Sainte Barbe, 1348 Louvain-la-Neuve, Belgium
* Correspondence: christian.bailly@uclouvain.be

Section S1 : Flash DSC Results

We use an ultrafast DSC to explore the melting characteristics of COPET4 after isothermal crystallization according to a "Tamman" method separating the nucleation and growth stages (see Section 2.7 of the manuscript for details).

Figure S1 shows the melting enthalpies and temperatures after crystallization at temperatures T_c ranging from 80°C to 150°C. Unsurprisingly, the maximum melting enthalpy (Figure S1a) is observed around 110°C T_c, halfway between the mobility limit imposed by T_g and the thermodynamic driving force limit imposed by T_m. Figure S1b shows the experimental T_m evolution as a function of T_c. The extrapolated intersection between this line and the $T_m = T_c$ diagonal is known to give an approximation of the thermodynamic melting point T_{m0}. Indeed, $T_m = T_c$ is the theoretical thermodynamic equilibrium situation of crystallization at zero undercooling. Although the extrapolation is not very precise, it is close to 290°C. This value is consistent with the thermodynamic melting point of neat PET reported in the literature by Metha [1]. The conclusion must be that the COPET crystals have the same thermodynamic characteristics as those of neat PET, hence the co-units are excluded from the crystals.

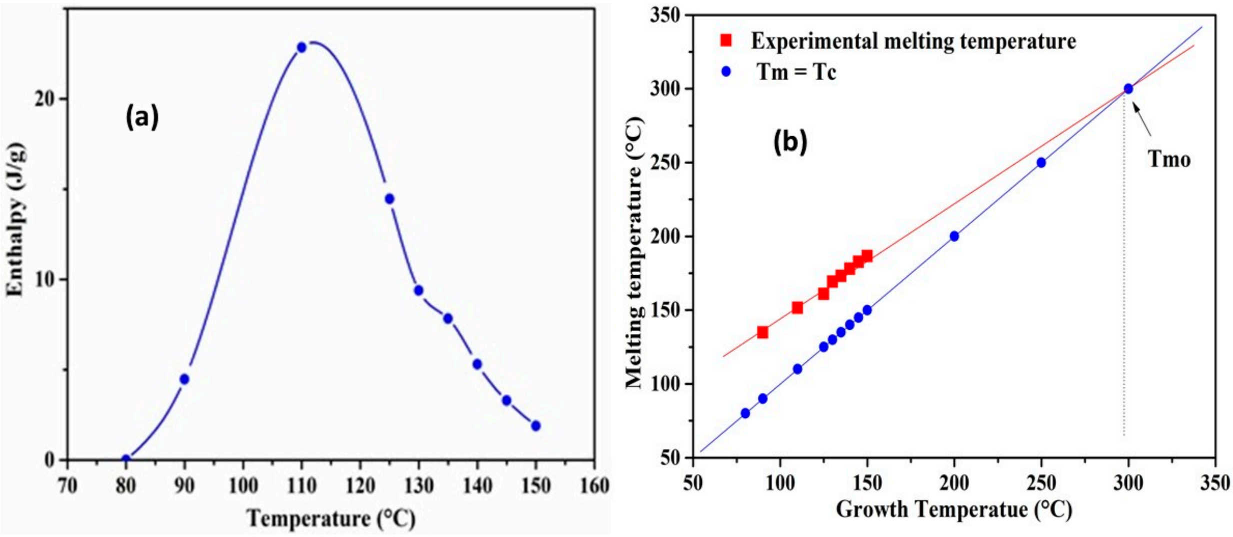

Figure S1. Melting enthalpy (**a**) and temperature (**b**) of the crystalline phase as a function of the crystal growth temperature for COPET4.

Section S2 : Banana Fibres Treatment

The alkaline treatment of the fibres is detailed in section II.5. The chemical functions before and after treatment were investigated by FTIR spectroscopy (Figure S2). The spectra have been normalized by matching the absorbance of the peak at 897cm^{-1} (C–O–C symmetric stretching of β-glycosidic ether band of the cellulosic material, which is unaffected by the treatment) [2]. The fibres treatment leads to significant differences between the infrared spectra as shown in Figure S2. The comparison between the spectra of untreated and treated banana fibres reveals that the intensity of the hydroxyl bond stretching at 3419 cm^{-1} increases after alkaline treatment. This is presumably the result of the breaking of hydrogen bonds between the –OH groups of cellulose and hemicellulose [3]. The peak at 2900 cm^{-1} is assigned to the –C–H stretching vibration from the -CH_2 group of cellulose and hemicellulose. The absorption peak around 1630 cm^{-1} is related to –C=O stretching of acetyl groups of hemicellulose and carboxyl aldehydes in lignin [4]. The large absorbance band between 1367 and 1319 cm^{-1} is assigned to methyl asymmetric (–CH_3) and methylene (–CH_2) symmetric deformations of lignin as observed for flax fibres [2][5]. The intensity of this peak is weaker after alkaline treatment of the banana fibres, indicating that some lignin has been removed by the treatment. In the region between 1367 and 1311cm^{-1} many absorption peaks are observed, corresponding to –OH undergoing in plane bending [6]. The peaks with high intensity at 1200 to 1000 cm^{-1} result from C–O–C asymmetric stretching (cellulose and hemicellulose) and C–O/C–C stretching vibrations.

The FTIR results thus confirm that the alkaline treatment contributes to remove a significant amount of low stability components such as pectin, lignin and partially hemicellulose. The alkaline treatment decreases the formation of intramolecular hydrogen bonds due to ionization of pectin carboxylic groups and increases interfacial bonding between fibres and matrix.

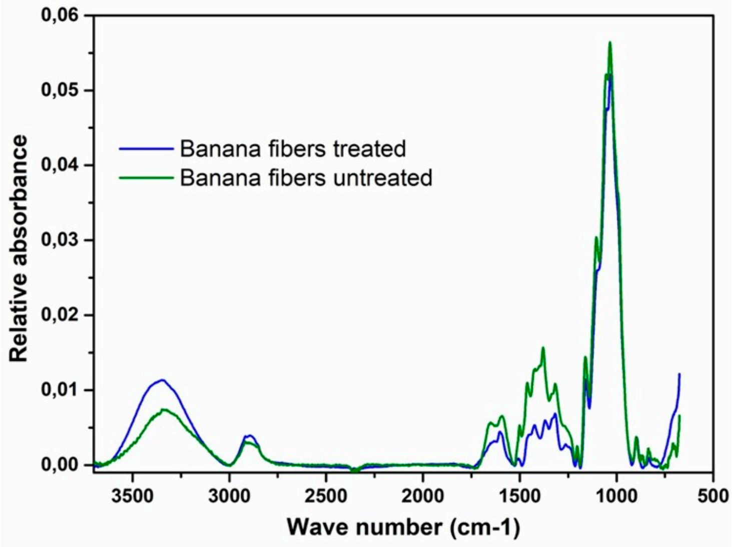

Figure S2. Influence of banana fibres alkaline treatment on FTIR spectra.

The physical properties of the fibres change upon heating in the range of 100 to 250°C. This can be explained by the alteration of physical or chemical structure by depolymerization, hydrolysis, oxidation, dehydration, decarboxylation, and recrystallization [7]. The thermal stability measured by TGA of untreated and treated banana fibres is presented in Figure S3. DTG (Figure S3b) reveals three peaks in the derivatives curve. The first one appears at 100°C (corresponding to 2–4% weight in loss in Figure S3a) and is related to the release of water. The second peak has a maximum degradation rate around 300°C for untreated banana fibres but 350°C for treated ones and corresponds to the degradation of cellulosic substances. The third peak occurring between 400 and 500°C is associated to the degradation of the non-cellulosic compounds. The remaining weight at weight stabilisation correlates with the percentage of residual ash. The treatment of banana fibres with alkaline solution thus increases the thermal stability by increasing temperature of the second degradation peak by 50°C, which is assigned to the removal of pectineus substances and to the corresponding increase of the cellulose content. The fibres will be only exposed to 170–200°C during melt processing of the copolyesters (composites) developed in this work while temperatures as high as 280°C are unavoidable in the case of neat PET.

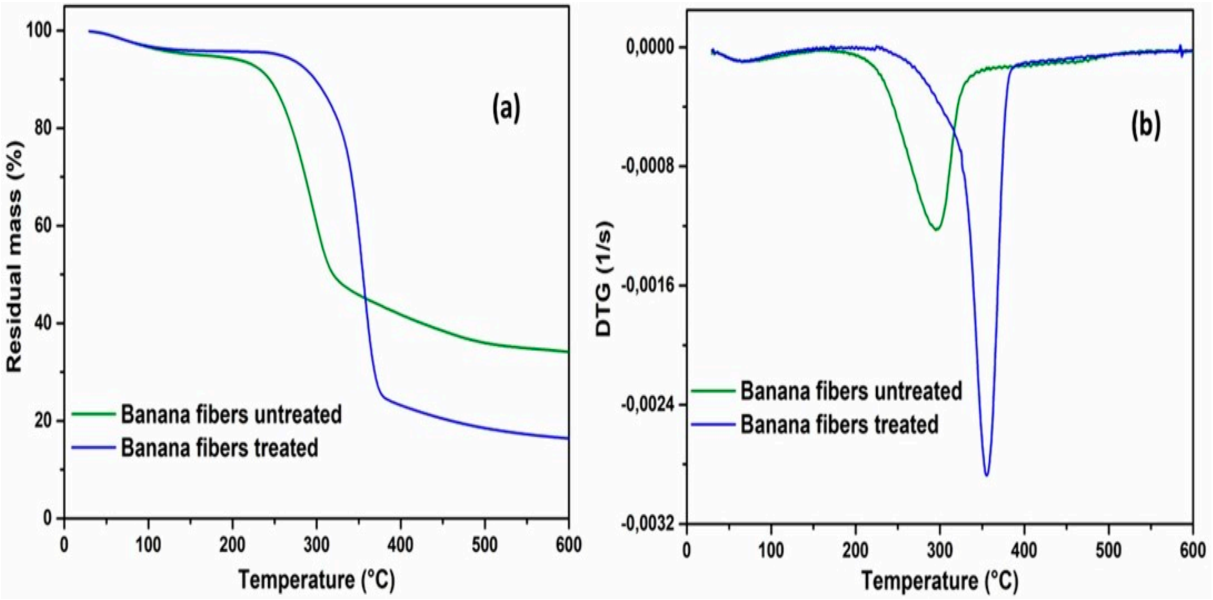

Figure S3. Influence of banana fibres treatment on thermal stability of banana fibres. (**a**)TGA and (**b**) DTG.

References

1. Mehta, A.; Gaur, U.; Wunderlich, B. Equilibrium Melting Parameters of Poly (ethy1ene Terephthalate). Polymer, **1978**,*16*, 289–296. Doi: 10.1002/pol.1978.180160209.
2. Cecci, R. R. R.; Passos, A. A.; Aguiar , N. T. C.; Silva, L. A. Banana pseudostem fibers characterization and comparison with reported data on jute and sisal fibers. *SN Appl. Sci.* **2020**, *2*, 1–6.
3. Saha, P.; Manna, S. ; Chowdhury, R.; Sen, R.; Roy, D. Adhikari, B. Bioresource Technology Enhancement of tensile strength of lignocellulosic jute fibers by alkali-steam treatment. *Bioresour. Technol.* ***2010***, *101*, 3182–3187. Doi: 10.1016/j.biortech.2009.12.010.
4. Haque, M.; Hasan, M.; Islam, S.; Ali, E. Bioresource Technology Physico-mechanical properties of chemically treated palm and coir fiber reinforced polypropylene composites. *Bioresour. Technol.* **2009**, *100*, 4903–4906. Doi: 10.1016/j.biortech.2009.04.072.
5. Bozaci, E.; Sever, K. ; Sarikanat, M. ; Seki, Y. ; Demir, A. ; Ozdogan, E. ; Tavman, I. Effects of the atmospheric plasma treatments on surface and mechanical properties of flax fiber and adhesion between fiber-matrix for composite materials. *Compos. Part B Eng.* **2013**, *45,* 565–572. Doi: 10.1016/j.compositesb.2012.09.042.
6. Parre, A.; Karthikeyan, B.; Balaji, A.; Udhayasankar, R. Proceedings Investigation of chemical, thermal and morphological properties of untreated and NaOH treated banana fiber. *Mater. Today Proc.* **2020**, *22*, 347–352. Doi: 10.1016/j.matpr.2019.06.655.
7. Gassan, J.; Bledzki, A. K. Thermal Degradation of Flax and Jute Fibers. *Appl. Polym. Sci*, **2001**, *82*, 1417–1422. Doi: 10.1002/app.1979.
